# Inductively Coupled Plasma Dry Etching of Silicon Deep Trenches with Extremely Vertical Smooth Sidewalls Used in Micro-Optical Gyroscopes

**DOI:** 10.3390/mi14040846

**Published:** 2023-04-14

**Authors:** Yuyu Zhang, Yu Wu, Quanquan Sun, Lifeng Shen, Jie Lan, Lingxi Guo, Zhenfeng Shen, Xuefang Wang, Junfeng Xiao, Jianfeng Xu

**Affiliations:** 1 State Key Laboratory of Digital Manufacturing Equipment and Technology, School of Mechanical Science and Engineering, Huazhong University of Science and Technology, Wuhan 430074, China; 2 Shanghai Aerospace Control Technology Institute, Shanghai 201109, China

**Keywords:** silicon deep trenches, Bosch process, pseudo-Bosch process, cryogenic etching

## Abstract

Micro-optical gyroscopes (MOGs) place a range of components of the fiber-optic gyroscope (FOG) onto a silicon substrate, enabling miniaturization, low cost, and batch processing. MOGs require high-precision waveguide trenches fabricated on silicon instead of the ultra-long interference ring of conventional F OGs. In our study, the Bosch process, pseudo-Bosch process, and cryogenic etching process were investigated to fabricate silicon deep trenches with vertical and smooth sidewalls. Different process parameters and mask layer materials were explored for their effect on etching. The effect of charges in the Al mask layer was found to cause undercut below the mask, which can be suppressed by selecting proper mask materials such as SiO_2_. Finally, ultra-long spiral trenches with a depth of 18.1 μm, a verticality of 89.23°, and an average roughness of trench sidewalls less than 3 nm were obtained using a cryogenic process at −100 °C.

## 1. Introduction

As the core of an inertial navigation system (INS), the gyroscope is responsible for providing angular velocity to assist in the calculation of velocity, position, and attitude information [1,2,3]. The interferometric fiber-optic gyroscope (IFOG) utilizes the Sagnac effect to measure rotational motions with light [4,5,6,7]. The interferometric ring inside the IFOG has two counterpropagating light beams with the same frequency, which will generate a phase difference as the IFOG rotates with the system, from which the angular velocity can be deduced. A longer interferometric ring is able to sense slighter motion, meaning a higher sensitivity of the gyroscope, but the resulting larger size limits further widespread applications [8,9,10]. With the development of optoelectronic and micro/nano processing technology, the miniaturization of optical gyroscopes became a major subject, and the micro-optical gyroscope (MOG) was launched [11,12]. The MOG relocates the entire set of FOG components, such as the photodetector, detection circuit, and fiber-optic waveguide, onto a substrate, typically silicon, integrating them into a monolithic device [12,13,14]. The migration of the ultra-long interference ring onto the silicon substrate is an essential part of the integration, as the accuracy of the ring directly affects the performance of the gyroscope [15]. Poor sidewall morphology of the silicon waveguide trenches can cause light wave loss when light passes through the trenches, thus decreasing measurement sensitivity and accuracy.

The fabrication technologies of silicon deep trenches include femtosecond laser processing [16,17,18], mechanical machining [19,20], and inductively coupled plasma (ICP) etching [21,22,23]. Femtosecond laser processing focuses a laser beam on the surface of a workpiece through an optical system, thereby melting and vaporizing the material to fabricate micro/nano structures. Crawford et al. [24] used 150 fs pulses with a central wavelength of 800 nm to machine silicon trenches up to 35 µm in depth, but with terrible verticality and roughness of sidewalls. Femtosecond laser technology works directly on the target material without additional masks, but it is not efficient for machining large-area microstructures due to the point-by-point scanning characteristic of the laser beam. The scanning speed of the laser beam used by Crawford is 0.5 mm/s max, clearly not applicable to the MOG waveguide, which may be over 30 m long. Xie et al. [25] attempted to process large areas, but SEM images showed that the uniformity of trenches was not satisfactory. Aurich et al. [20] investigated microgrinding to fabricate trenches with a roughness of only 10 nm. However, this method, depending on the tool’s geometry, makes it difficult to machine high aspect ratio trenches. Meanwhile, the continuous cutting workload damages the tools significantly and is unsuitable for batch production.

ICP etching manufactures various semiconductor micro/nano structures on the substrates, such as Si, SiO_2_, SiC, and diamond, by sputtering the substrate with plasma generated from ionized appropriate gas (glow discharge), which is widely used in integrated circuits, MEMS, optics, etc. [26,27,28]. ICP etchers normally contain two radio frequency (RF) sources with a frequency of 13.56 MHz, serving as an ICP generator and a capacitively coupled plasma (CCP) generator, respectively. During ICP etching, the chosen gas flowing into the working chamber is ionized by the top ICP generator to produce numerous plasma-containing charged particles, radicals, and neutral atomic molecules [29,30,31]. The reactive plasma bombards the wafer vertically downward, accelerated by a DC bias voltage applied from the bottom CCP generator (RF power), enabling removal of substrate material physically and chemically. ICP etching is commonly accompanied by a lithography process, leading to complex operating procedures. However, the advantage of its simultaneous molding structure makes it suitable for the fabrication of large-area complex microstructures, such as the ultra-long spiral trench in this experiment. Gerlt et al. [32] presented an optimized three-step Bosch process, allowing the fabrication of structures with 6μm width at depths up to 180μm, but the researchers did not attend to the sidewall roughness. Michael [33] used the pseudo-Bosch process to machine silicon pillars with a minimum surface roughness of 5 nm but only 800 nm in depth. These show that manufacturing deep microstructures with smooth surface and vertical sidewalls remains challenging.

In this paper, we explored different etching methods to achieve deep silicon trenches with high verticality and low roughness. Photoresist, Al, and SiO_2_ were selected as masks for different etching processes. A Partial Least Squares (PLS) regression model was proposed to investigate the relationship between process parameters and the etching profile.

## 2. Materials and Methods

### 2.1. Materials

The silicon wafers from RDMICRO company (Suzhou, China) are 6-inch, crystal phase <100>, and 1500 μm thick. All silicon samples were cleaned sequentially with acetone, isopropanol, and anhydrous ethanol by ultrasonication for 3 min and baked at 100℃ for 10 min before the experiment. AZ4620 is a thick photoresist (PR) used as a soft mask during the Bosch process, and AZ5214 is a thin PR used in preparation of hard mask layers. The developer AZ400K was diluted with deionized (DI) water at 1:4 for use. The three materials above were sourced from AZ Electronic Materials (Luxembourg). The N-Methylpyrrolidone (NMP) solution (99.9% purity) dissolved the photoresist for the purpose of removal and was purchased from Shanghai Aladdin (Shanghai, China).

### 2.2. Experimental Equipment

In this experiment, electron beam evaporation deposition (F.S.E. Corporation. FU-121, New Taipei, China) was used to plate Al films. The SiO_2_ films on silicon wafers were grown on a PECVD device (Oxford, Plasma Pro System 100, Yatton, UK). The Bosch process and the pseudo-Bosch etching were carried out on the Plasma lab system 100 ICP 180 (Oxford, Yatton, UK), while the cryogenic etching process was investigated on the Estrelas 100 (Oxford, Yatton, UK). The tray in the working chamber of the latter is connected to a liquid nitrogen cooling system for cryogenic control of wafers. 

### 2.3. Methods

#### 2.3.1. Geometry Design of MOG Waveguide Trenches

**Figure 1:**
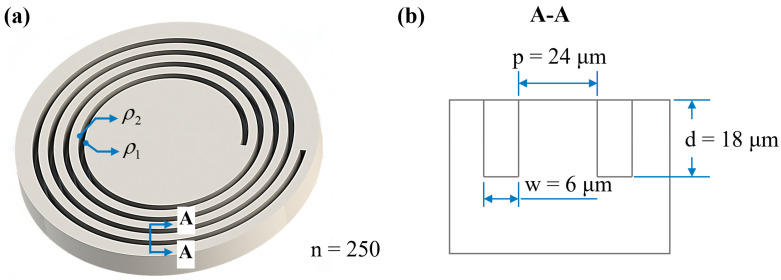
MOG spiral trenches: (**a**) 3D structure diagram and (**b**) geometry design.

The MOG waveguide adopted the Archimedean spiral structure, satisfying both ultra-long length and low footprint. Also, the Archimedean spiral, known as the arithmetic spiral, ensures a constant trench width and pitch between adjacent trenches. The designed dimensions of the trench are 6 μm wide, 18 μm deep, and 24 μm apart, as shown in Figure 1. The spiral ring has a total length of 37.34 m with 250 laps (*n* = 250), using generated path data by MATLAB R2019a and drawn by AutoCAD 2020 and SOLIDWORKS 2016. The polar equations for the inner ring $\rho_{1}$ and outer ring $\rho_{2}$ are as follows (in mm):

(1) $$\left\{\begin{array}{l} \rho_{1}=20+\frac{0.015}{\pi }*\theta \\ \rho_{2}=20.006+\frac{0.015}{\pi }*\theta \end{array}\right.\;,\;\theta \in [0,2\pi *250]$$

#### 2.3.2. ICP Etching of MOG Spiral Trenches

The three processes considered in this experiment, i.e., the Bosch process, pseudo-Bosch process, and cryogenic etching, are different in terms of the etchant gas type and the entry sequence into the reactor chamber, resulting in variations in their etching mechanisms.

The Bosch process, with SF_6_ as the etching gas and C_4_F_8_ as the passivation gas used alternately in the reactor chamber, cycles the etching and passivation steps for deep silicon etching [34,35,36,37]. During the passivation cycle, C_4_F_8_ is ionized by ICP power and deposited on all exposed surfaces forming a fluorocarbon polymer to prevent erosion. In the subsequent etching period, SF_6_ mainly ionizes into SF_x_ (x = 1, 2, 3, 4, and 5) and F ions, which are attracted by the bias voltage out of the plasma ionization region above the chamber and accelerated downward to bombard the substrate [38,39]. The SF_x_ ions physically sputter to remove the passivation polymer layer at the trench bottom, and F^-^ subsequently reacts with the exposed silicon to deepen the trench. As the cycles continue, the trench depth increases, while the sidewalls remain vertical, thanks to the presence of the passivation film. In the pseudo-Bosch process, SF_6_ and C_4_F_8_ are injected simultaneously; therefore, etching and passivation are performed together [40,41]. The cryogenic process operates below −100 °C and adopts O_2_ as the passivation gas, working concurrently with SF_6_. O_2_ ionizes to form O ions, which combine with Si and F atoms to deposit SiO_x_F_y_ on the trench sidewalls to reduce over-etching. The SiO_x_F_y_ polymer will decompose automatically as the reactor chamber recovers to room temperature after etching [42,43,44,45].

**Figure 2:**
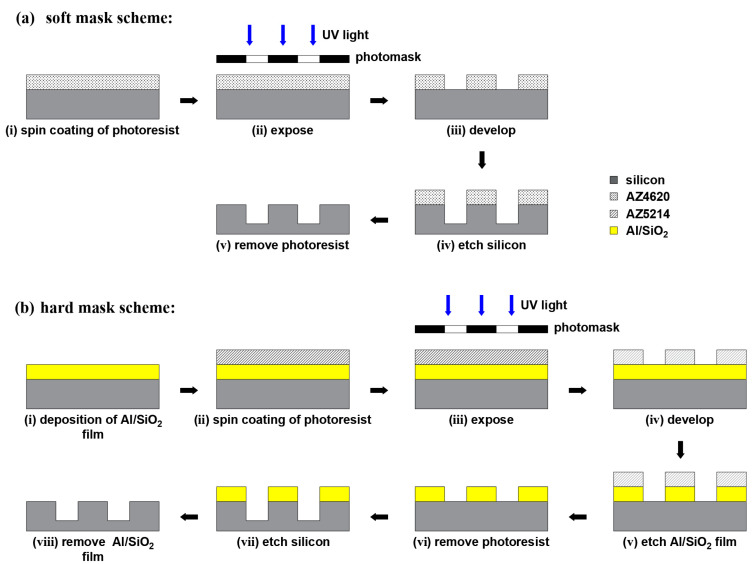
Schematic illustration of ICP etching MOG spiral trenches: (**a**) soft mask scheme and (**b**) hard mask scheme.

The Bosch process has a low etch rate for photoresists, i.e., a high selectivity (the ratio of the etching rate of silicon to the mask layer), as the result of alternating etching and passivation. Photoresists can be directly used as mask layer material for the Bosch process, as illustrated in Figure 2a. After spin-coating photoresist on the wafer surface (Step i), the spiral pattern on the photomask was transferred to the soft mask layer by lithography (Step ii, iii). During etching (Step iv), the area covering AZ4620 on the wafer was not etched; instead, the silicon material in the uncovered area was continuously removed to form trenches, eventually removing the photoresist by NMP solution. For the pseudo-Bosch and cryogenic processes, etching and passivation are running in parallel, and thus the etching rate of the photoresist becomes greater, which possibly causes the problem of depleting the mask layer before the etching step is finished. Consequently, the pseudo-Bosch and cryogenic processes utilized Al and SiO_2_ as hard mask layer materials separately (shown in Figure 2b). An additional etching process (Step v) was required for transferring the target pattern to the hard mask layer before etching the silicon (Step vii). Al film was etched with Cl_2_/BCl_3,_ and SiO_2_ with CHF_3_/Ar. After the whole etching process was complete, the Al film was stripped with a boiled sulfuric acid/peroxide mixture (SPM), and SiO_2_ was stripped with buffered hydrofluoric acid (BHF).

### 2.4. Characterization

**Figure 3:**
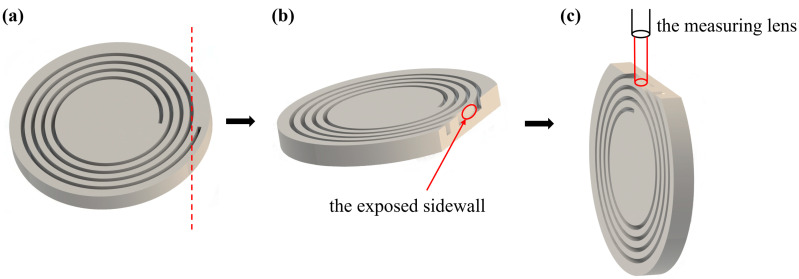
Measurement illustration of sidewall roughness: (**a**) cutting the sample, (**b**) the sidewalls of marked area were completely exposed, allowing roughness characterization by white light interferometer, and (**c**) the sample was placed vertically under the measuring lens of the white light interferometer, which was then adjusted to focus on the exposed sidewall area for roughness measurement.

The film thickness of SiO_2,_ Al, and PR, respectively, was measured by a spectroscopic ellipsometer (Semilab, GES5E, Budapest, Hungary) and a stylus profilometer (Bruker, DektakXT, Billerica, MA, USA). All SEM images were obtained by scanning electron microscopes (Hitachi, SU3900, Tokyo, Japan and FEI, Quanta 3D FEG, Hillsboro, OR, USA), and the surface morphology of MOG spiral trenches was observed by a metallographic microscope (Zeiss, Axiocam 208 color, Jena, Germany). A white light interferometer (Zygo, NewView 9000, Middlefield, CT, USA) was used to characterize the surface roughness of sidewalls after cutting the sample tangentially along the trench sidewall (illustrated in Figure 3).

## 3. Results and Discussion

### 3.1. Bosch Process

**Figure 4:**
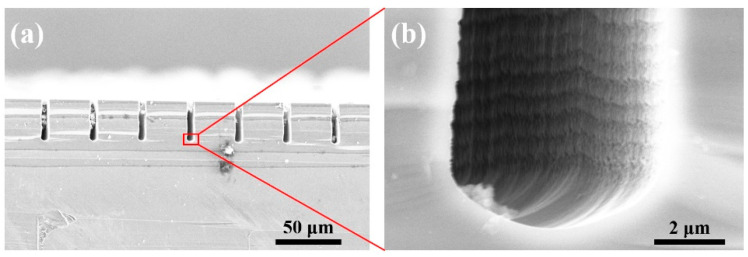
SEM images of (**a**) trenches and (**b**) sidewall of micro trenches by Bosch process.

As previously mentioned, the etching gas SF_6_ and passivation gas C_4_F_8_ are alternated in the Bosch process. To ensure process stability and repeatability, each gas was applied in small amounts during each etching or passivation cycle. SF_6_ was injected during deposition, and C_4_F_8_ during etching, both with a flow of 5 sccm. The soft mask layer AZ4620 was 6 µm thick (4000 rpm), and the optimized process parameters are listed in Table 1. After 100 cycles (30 min in duration), silicon trenches of 30.72 µm deep were obtained, with an etching rate of 1.02 µm/min. Although the trench depth was higher than 18 µm, which could be adjusted by reducing the number of cycles (total cycle time), the critical problem was the scalloped undulation of the sidewalls, as shown in Figure 4.

It is the result of lateral erosion acting on the newly etched sidewalls without the protection of passivation film during the etching cycle, leading to continuous undulations as the cycles persist. The size of the scallop microstructures was around 70–80 nm, not achieving low sidewall roughness.

### 3.2. Pseudo-Bosch Process

**Figure 5:**
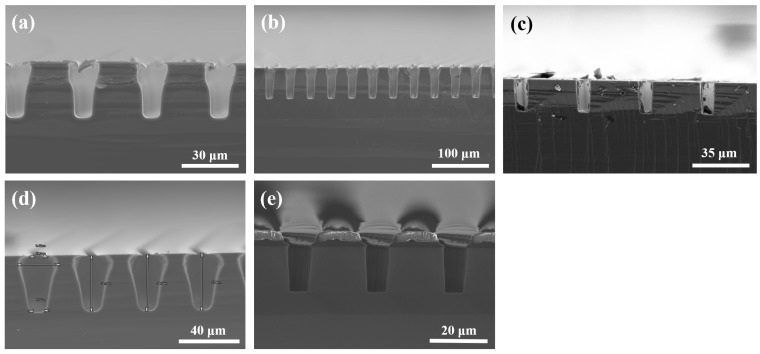
Different profiles of pseudo-Bosch experiments in (**a**) Group 1, (**b**) Group 2, (**c**) Group 3, (**d**) Group 4, and (**e**) Group 5.

Since the switching between etching and passivation caused scallops on the sidewalls of deep trenches, the pseudo-Bosch process was attempted instead. Separate passivation before etching in the Bosch process guarantees minimal corrosion of the soft mask layer, but when carried out together, the etching rate increases significantly. Metal films are highly resistant to fluorine-based plasma etching and have a good selectivity relative to silicon substrates, making it sufficient for use as a hard mask with only a thin film. A 400 nm thick Al film was therefore chosen as the hard mask for the pseudo-Bosch process in view of its simplicity for deposition by evaporation and ease-of-use for mask patterning by chlorine plasma etching. The experimental data and results are detailed in Table 2, and Figure 5 shows the SEM images of trench sections using different parameters in the pseudo-Bosch process. Groups 1–3 changed the flow of C_4_F_8,_ and Group 4 used the low flow of SF_6_ and C_4_F_8_ for verifying the effect of plasma density.

It can be seen in Figure 5 that lateral etching occurred on the silicon beneath the mask, especially on the top sidewalls more severely, called undercut. Through searching literature and analysis, it was determined to be caused by the metal mask. The eddy current heating effect [46] and charges in the mask layer [47,48] are responsible for this result. For conductive metal masks, the RF power generates eddy currents inside the metal mask, causing heating that contributes to the desorption of the surrounding fluorocarbon film and thus speeding up the etching below the Al mask. But the heat flux caused by eddy currents is minor and not sufficient to dominate. The positive charge at the bottom of the Al mask deflects the CF_x_^+^ ions that undertake a major role in the passivation deposition [49], reducing the thickness of the passivation layer at the top sidewall, which leads to increased lateral etching of this region. Figure 5a–c reveal that undercut was suppressed with the increase of passivation gas C_4_F_8_ flow, confirming that this defect is mainly controlled by the passivation stage. An additional group that reduced the flow of both gases, as displayed in Figure 5d, also occurred with undercut, excluding the disturbance of higher ion densities due to high fluxes in Groups 1–3. 

Finally, SiO_2_ was determined as a reliable hard mask material. The thickness of the SiO_2_ layer prepared by PECVD was around 2 µm, and the final process parameters are presented as the fifth recipe in Table 2. The undercut under the mask was alleviated as the mask material changed to SiO_2_ (as shown in Figure 5e), and the etching rate was reached at 1.62 μm/min, but the verticality of the sidewall remains to be improved. 

### 3.3. Cryogenic Etching

Although the pseudo-Bosch process using SiO_2_ as a mask layer has been proven to achieve micro-trenches with good morphological sidewalls, the etching rate is still undesirable. For further improvement, we investigated cryogenic etching for deep trenches. SF_6_ and O_2_ are used for etching and passivation, respectively, in which SF_6_ provides F radicals to react with silicon, and meanwhile, O_2_ combines with other particles to generate SiF_x_O_y_ film, the passivation layer in the cryogenic etching process.

In this experiment, SiO_2_ remained as the hard mask layer, and the working temperature of cryogenic etching was set to −100 °C. The work aimed to obtain vertical and smooth sidewalls by varying the flow of O_2_ and the process time, keeping ICP power, RF power, temperature, and chamber pressure constant (as shown in Table 3).

In both Bosch and pseudo-Bosch processes, the high-energy plasma bombardment and etching of the silicon wafer increase the substrate temperature, contributing to non-uniformity of etching. The cryogenic process allows the wafer to be connected with a liquid nitrogen cooling system, which provides a consistent cryogenic environment over the entire wafer, thereby improving the etch profile. 

**Figure 6:**
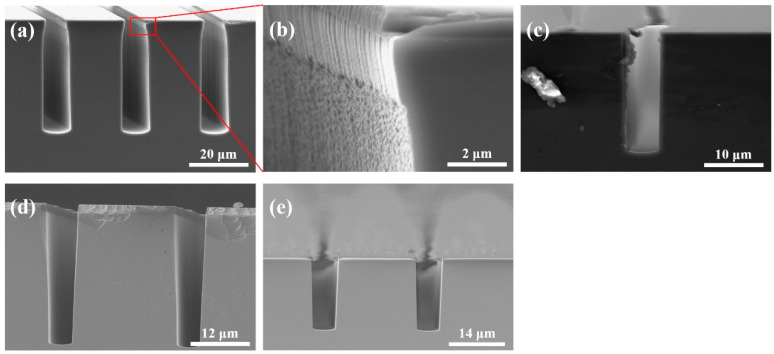
Etching results of cryogenic etching with different O_2_ flows and process times: (**a**) Group 1: O_2_ flow = 8 sccm, time = 10 min, (**b**) local enlargement of the sidewall of Group 1, (**c**) Group 2: O_2_ flow = 8.5 sccm, time = 4 min, (**d**) Group 3: O_2_ flow = 10 sccm, time = 6 min, (**e**) Group 4: O_2_ flow = 8 sccm, time = 3.5 min.

The results of cryogenic etching are concentrated in Figure 6. With an oxygen flow of 8 sccm, a depth of 32.81 µm can be reached after 10 min of etching. But from its SEM image (as shown in Figure 6a), the profile is observed as narrow at the top and wide at the bottom, resembling a bottle, and the magnified view of sidewalls shows an uneven morphology, measured to undulate at 40–60 nm. The trench profile evolved towards a V-shaped structure as O_2_ flow increased (Figure 6d), and the undulations on the sidewalls gradually disappeared. This can be summarized as the increase in O_2_ flow improves passivation efficiency and enhances the protection of sidewalls. It was also found that the etching rate should theoretically decrease with increasing O_2_ flow, but the data from the previous three groups are controversial, indicating that the average etching rate is also related to the process time. For this, a fourth group was built as the comparison group for the first group, with identical parameters, except the process time was shortened to 3.5 min. The differences in etching rates and profiles between these two groups confirmed our guess. We speculate that the gradual deepening of the trench and the property of the high aspect ratio of the trench contribute to the reduced efficiency of the plasma in reaching the trench bottom and removing the silicon material, intuitively expressed as an effect of process time, i.e., the shorter the process time, the faster the average etching rate.

A Partial Least Squares (PLS) regression model was created to analyze the correlation between gas flow, process time, and etching profile. The SF_6_ to O_2_ ratio and the process time were selected as the independent variables *x*_1_ and *x*_2_, and the etching depth and tilt angle as the dependent variables *y*_1_ and *y*_2_. Based on the experimental data in Table 3, the regression model was established as follows:

(2) $$\left\{\begin{array}{l} y_{1}=21.8475-0.9558x_{1}+0.0371x_{2}\\ y_{2}=82.1027+0.4329x_{1}+0.0078x_{2} \end{array}\right.$$

**Figure 7:**
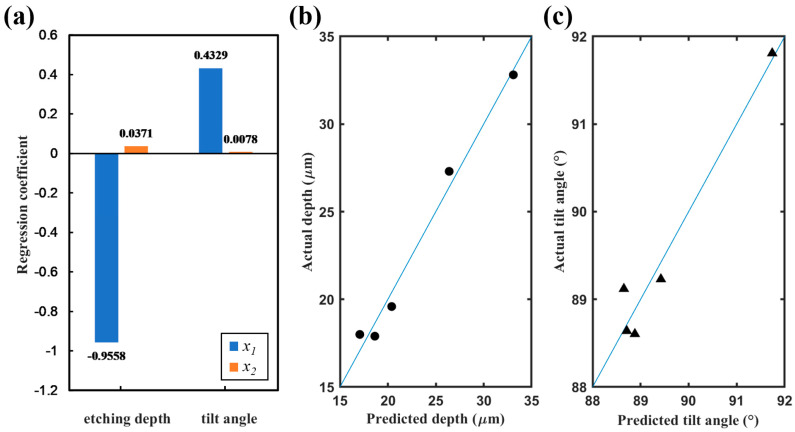
PLS regression model of cryogenic etching: (**a**) histogram of regression coefficients, (**b**) prediction graph of etching depth, and (**c**) prediction graph of tilt angle.

**Figure 8:**
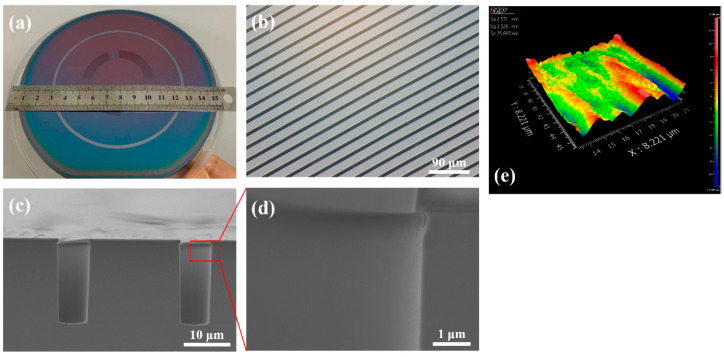
The images of final spiral trench sample: (**a**) picture of the entire sample, (**b**) surface morphology of MOG spiral trenches, (**c**) SEM image of trench profile, (**d**) partial magnification of the sidewall, and (**e**) roughness image of the sidewall.

The histogram of the regression coefficients (shown in Figure 7a) visualizes that the regression coefficients of the ratio of SF_6_ to O_2_ flow *x*_1_ is much larger than the process time *x*_2_ for both etching depth *y*_1_ and tilt angle *y*_2_, meaning that the gas flow has a more significant effect on the etching profiles. Figure 7b and c, respectively, display the prediction graphs of etching depth and tilt angle based on the regression equation, and the horizontal and vertical coordinates of data points separately represent the fitted values and the actual values. Data points closer to the diagonal line mean that the predicted value is closer to the actual value, implying the higher confidence of the regression equation. Compared with Figure 7c, the fitted results of Figure 7b about the etching depth are better, indicating the regression equation of the etching depth is more desirable, which is because the regression coefficients of the tilt angle are both lower than those of the etching depth. Overall, the minor differences between the fitted values of the equation and the original data demonstrate that the model created is acceptable. According to the model, for obtaining a trench with 90° sidewalls, the gas ratio should be 13.1, namely an O_2_ flow of 7 sccm, as in the fifth recipe in Table 3. After successive explorations, the desirable silicon spiral trench sample is displayed in Figure 8, with a sidewall verticality of 89.2°, an average width of 6.1 µm, and an average depth of 18.1 µm. Moreover, the average roughness of the trench sidewall characterized by the white light interferometer is only about 2.5 nm, which is significant for future light wave conduction.

## 4. Conclusions

In this study, the Bosch process, pseudo-Bosch process, and cryogenic etching were used to fabricate silicon spiral deep trenches. The Bosch process, with its inherent characteristic of alternate etching and passivation, could produce 70–80 nm scallops on the sidewalls of the deep trenches. Undercut of the silicon beneath the Al mask occurred in the pseudo-Bosch process. The charges in the mask are responsible for this result, achieved by interference passivation, and this negative effect was mitigated by replacing Al with SiO_2_ as the hard mask. A cryogenic process with a higher etching rate was demonstrated as a viable method in which cryogenic control ensures the stable temperature of the wafer during the process and etching uniformity. It was found that the etching rate of the deep trenches was also dependent on the process time. The association of the etching rate with gas flow and process time was revealed by a PLS regression model. The optimal silicon trench with a verticality of 89.23° was fabricated, and the sidewall roughness was only 2.5 nm on average. Our future work includes the selection and deposition into silicon trenches of optical waveguide materials, the construction of optical platforms for measuring light wave transmission, and the combination of silicon trenches with an MOG.

## Figures and Tables

**Table 1:** Bosch process etching parameters.

	ICP Power (W)	RF Power (W)	Gas Flow (sccm)		Temperature (°C)	Pressure (mTorr)	Time (s)
			SF_6_	C_4_F_8_			
Etching	700	25	100	5	10	40	8
Passivation	700	10	5	100	10	30	10
Cycles							1800

**Table 2:** Pseudo-Bosch process etching parameters and results.

Number	ICP Power (W)	RF Power (W)	Gas Flow (sccm)		Temperature (°C)	Pressure (mTorr)	Time (min)	Etching Rate (μm/min)
			SF_6_	C_4_F_8_				
1	1200	20	33	33	15	10	23.2	1.22
2	1200	20	33	57	15	10	114	0.48
3	1200	20	33	72	15	10	70	0.31
4	600	30	12	15	15	10	45	0.84
5	1000	45	100	120	5	15	10	1.62

**Table 3:** Cryogenic process etching parameters and results.

Number	ICP Power (W)	RF Power (W)	Gas Flow (sccm)		Temperature (°C)	Pressure (mTorr)	Time (min)	Etching Rate (μm/min)	Tilt Angle (°)
			SF_6_	O_2_					
1	600	8	92	8	−100	12	10	3.28	91.8
2	600	8	92	8.5	−100	12	4	4.90	89.1
3	600	8	92	10	−100	12	6	4.55	88.6
4	600	8	92	8	−100	12	3.5	5.12	88.6
5	600	8	92	7	−100	12	3.5	5.14	89.2

## Data Availability

Data sharing is not applicable to this article.
